# Wound Segmentation with U-Net Using a Dual Attention Mechanism and Transfer Learning

**DOI:** 10.1007/s10278-025-01386-w

**Published:** 2025-01-23

**Authors:** Rania Niri, Sofia Zahia, Alessio Stefanelli, Kaushal Sharma, Sebastian Probst, Swann Pichon, Guillaume Chanel

**Affiliations:** 1 Computer Science Department, University of Geneva, Geneva, Switzerland; 2 Institute of Industrial and IT Engineering, HEPIA, HES-SO Geneva University of Applied Sciences and Arts, Western Switzerland, Geneva, Switzerland; 3 School of Health Sciences, HES-SO Geneva University of Applied Sciences and Arts, Western Switzerland, Geneva, Switzerland; 4 Care Directorate, Geneva University Hospitals, Geneva, Switzerland; 5 imito AG, Zurich, Switzerland

**Keywords:** U-Net, Attention networks, Wound segmentation, Medical imaging, Deep learning

## Abstract

Accurate wound segmentation is crucial for the precise diagnosis and treatment of various skin conditions through image analysis. In this paper, we introduce a novel dual attention U-Net model designed for precise wound segmentation. Our proposed architecture integrates two widely used deep learning models, VGG16 and U-Net, incorporating dual attention mechanisms to focus on relevant regions within the wound area. Initially trained on diabetic foot ulcer images, we fine-tuned the model to acute and chronic wound images and conducted a comprehensive comparison with other state-of-the-art models. The results highlight the superior performance of our proposed dual attention model, achieving a Dice coefficient and IoU of 94.1% and 89.3%, respectively, on the test set. This underscores the robustness of our method and its capacity to generalize effectively to new data.

## Introduction

Skin wounds have a substantial negative impact on patients’ health and quality of life, and represent a substantial economic burden to the healthcare system worldwide. Recent reports indicate that close to one billion individuals worldwide experience both acute and chronic wounds [1, 2]. Acute wounds can heal within a predictable period of time depending on the nature of the injury whereas chronic wounds such as diabetic foot ulcers (DFU), leg- or pressure ulcers fail to resolve and are characterized by pathological processes, such as continuous inflammation, persistent infections, and necrosis [3]. The management and treatment of such wounds are challenging, requiring a systematic and multidisciplinary approach to the patient, their comorbidity, and the wound itself [4]. Clinical practice shows that each clinician may hold diverse and unique perspectives on wound therapy based on their prior experiences. Nonetheless, every one of these healthcare providers is delivering wound care. It is therefore important to push efforts into the standardization of wound monitoring and management. An accurate wound assessment includes a comprehensive patient history, etiology of the wound, the wound characteristics such as size, depth, condition of the wound bed and peri-wound area including the amount, color, and consistency of exudate as well as signs of infection [5].

Standard clinical practice for wound examination mainly involves techniques to measure the wound dimensions, such as measuring the perimeter with a ruler or outlining the wound on a transparent sheet to calculate surface area. These methods are highly subjective, time-consuming, and are prone to errors. Alternatively, medical imaging technology based on artificial intelligence (AI) and deep learning techniques (DL) has increasingly become a common practice for automatic assessment of chronic wounds in clinical settings in recent years. This shift has brought about radical changes in wound care practices. Unlike the measurement methods mentioned above, techniques based on AI and automatic image analysis offer the potential to reduce the risk of infection while improving the accuracy, speed, and objectivity of wound diagnosis. Additionally, AI and DL helped reduce the burden on healthcare professionals by automating wound assessment routines. Thus, providing clinicians with valuable information regarding wound progression over time, allowing them to have more time to focus on more complex aspects of the wound management. This collaborative effort between technology and healthcare professionals can lead to enhanced outcomes for medical practitioners and patients alike.

The first step towards an effective automated image analysis is to provide efficient algorithms for segmenting the wound area or what we call the region of interest (ROI). This step aims to identify and separate the ROI from the surrounding healthy tissue while eliminating all background elements in an image. The segmentation of wound images can be a challenging task due to their variability in size, shape, texture, and color because of the presence of different tissue types, as well as the presence of artifacts and noise in the images. Nonetheless, the accurate segmentation of these images is crucial for effective wound management, as it enables clinicians to accurately measure and track the healing progress over time.

Recent advances in DL have enabled the development of powerful semantic segmentation algorithms for wound image analysis providing reliable and promising results. DL semantic segmentation algorithms use fully convolutional neural networks (FCNs) to process images and generate pixel-wise output maps that classify each pixel in the image as belonging to the wound or non-wound class. The U-Net architecture [6] is a widely used FCN architecture for medical image segmentation tasks, as it has demonstrated high performance and accuracy in a variety of applications including wound image segmentation. Its versatility and performance have made it a popular choice among researchers in the field.

However, there are also several challenges associated with the use of DL for wound segmentation. First, in the case of wound images, collecting a large dataset with accurate ground truth segmentation masks can be difficult and time-consuming. Also, wounds show considerable variability, presenting a challenge for DL algorithms to generalize on new, unseen wound images. Finally, wound segmentation in a clinical environment remains challenging due to lighting conditions, noise, and artifacts such as shadows, reflections, and debris, which can interfere with the segmentation process and lead to inaccurate results and require additional post-processing steps.

This paper proposes a novel wound segmentation architecture using U-Net and a dual attention mechanism comprising attention gates (AG) [7] and squeeze-and-excitation (SE) blocks [8]. In the proposed approach, attention gates are used to highlight the most important features in the image, while SE blocks are used to recalibrate the feature maps to emphasize the most informative channels. By combining these two powerful techniques, the network can benefit from both spatial and channel-wise feature selection, which can enhance the accuracy of the segmentation by reducing the impact of noise and irrelevant information in the input. Additionally, the improved U-Net architecture was built upon a pre-trained VGG16 model backbone [9] to further improve the performance and reduce the computational cost.

The aim of this study is to evaluate the performance of the proposed model in terms of segmentation accuracy and compare it with other state-of-the-art models. We conducted extensive experiments on several databases, focusing on diabetic foot ulcers because of their chronic wound characteristics, ischemia, and infection [10]. Our method provides accurate and reliable segmentation, making it suitable for wounds of various types and sizes, regardless of their location or healing stage.

## Related Work

### Wound Segmentation

One of the earliest works in this domain was conducted by Wang et al. [11], where the authors introduced a deep learning encoder-decoder architecture for wound segmentation. The training was performed on a dataset of 650 images from the NYU database [12]. The achieved IoU score was of 47.3% indicating a modest performance. Goyal et al. [13] took a different approach and compared various Fully Convolutional Network (FCN) architectures for automated segmentation of diabetic foot ulcers (DFU) and surrounding skin using a dataset of 705 images. Their findings showed that FCN-16 s outperformed other architectures, achieving dice scores of 79.4% for ulcer regions and 85.1% for the surrounding skin.

In an attempt to improve segmentation quality, Li et al. [14] developed a system based on MobileNet using 950 wound images. Employing a preprocessing step involving watershed and dynamic thresholding, the authors aimed to simplify the segmentation task and enhance network performance. As a result, they obtained an average IoU index of 85%. A two-stage wound segmentation pipeline was later proposed by [15], which comprised wound segmentation and skin detection to overcome complex background elimination in clinical environments. By combining skin and wound segmentation masks, the authors achieved an impressive Dice score of 99.26% and an IoU of 98.48%. However, this method relied on a relatively small dataset of 569 images, requiring additional skin annotations.

To address the challenge of limited data, Mahbod et al. [16] proposed an ensemble of LinkNet and U-Net networks with EfficientNetB1 and B2 pretrained weights. Various augmentation techniques were applied, resulting in an average Dice Similarity Coefficient of 88.80%. Gamage et al. [17] compared Mask-RCNN, Faster RCNN with Mask-RCNN extension, and U-Net for ulcer segmentation using 400 ulcers. Despite employing an offline data augmentation approach to expand the training dataset, the Mask-RCNN model with a ResNet-101 backbone achieved only an average precision of 50.84%, which is considered to be a suboptimal performance. Another study by Zahia et al. [18] also used Mask-RCNN with a ResNet-50 backbone to segment wound areas. A mean Dice score of 83% was achieved. However, this method was tested on a small dataset of 200 images.

**Fig. 1:**
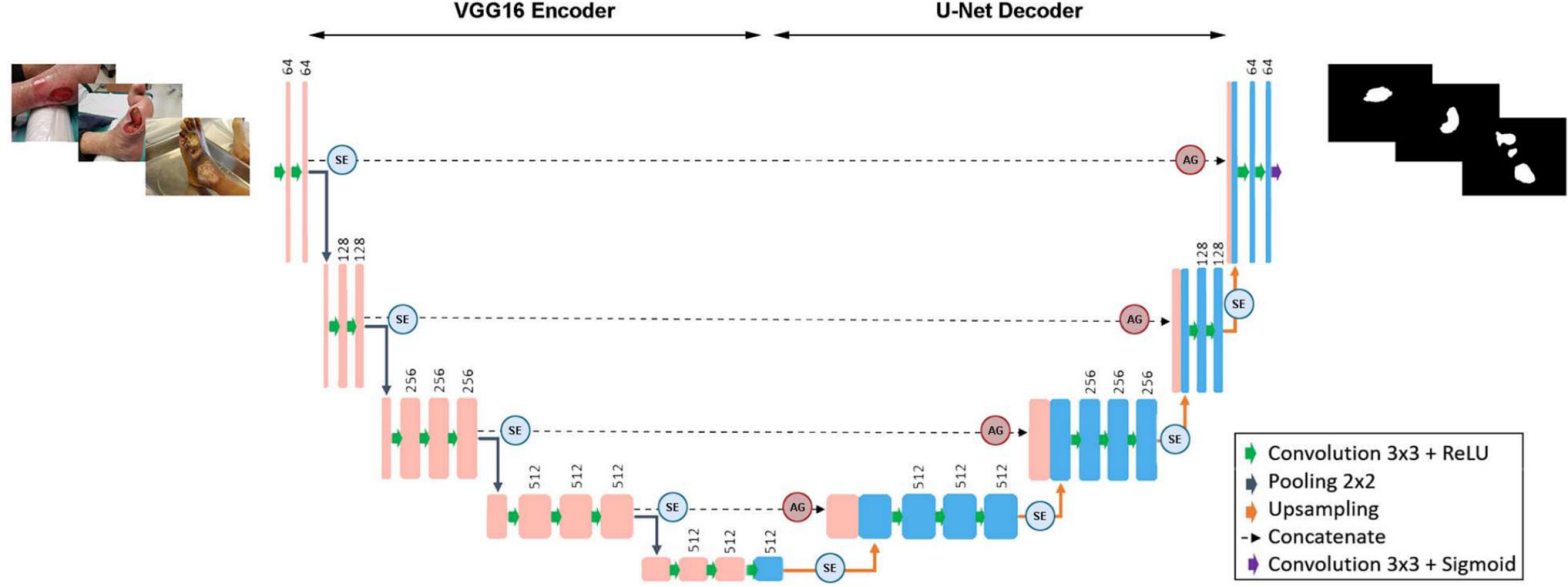
Overview of the proposed dual attention VGG6-UNet

More recently, the dataset released during the DFUC2022 Challenge has prompted the development of various deep learning methods for diabetic foot ulcer segmentation. The published papers offer unique contributions to the field, exploring different challenges of DFU segmentation and proposing novel solutions. The baseline paper by Kendrick et al. [19] introduced a patch-based framework using FCN32 with a VGG backbone, achieving first place in the challenge with an impressive Dice score of 72.87%. Similarly, Chen et al. [20] presented a novel patch-based approach using higher-resolution images. By dividing images into smaller patches, performing segmentation on each patch, and then reassembling the results through an ensemble approach, the method effectively captures fine-grained details, leading to higher accuracy and improved performance. However, relying solely on patches may limit the model’s ability to capture larger-scale patterns and contextual information.

Some papers focused on enhancing existing architectures and backbones to improve efficiency and boundary detection capabilities. HarDNet-DFUS [21] enhanced the backbone and decoder of the HarDNet-MSEG introduced in [22]. The method shows promising results, particularly in capturing intricate details within the ulcers. However, the segmentation performance may still be affected by issues like class imbalance. The authors in [23], introduced edge loss, leading to improved preservation of lesion boundaries of OCRNet [24]. This approach enhances boundary detection, leading to more precise segmentation results compared to the original network.

Scebba et al. [25] conducted a comparative study of various state-of-the-art segmentation models, including U-Net, ConvNet, DeepLabV3 (ResNet-101 backbone), and FCN (VGG16 backbone). While their approach highlighted strengths and weaknesses across models, their pipeline involved a multi-step process comprising wound detection, post-processing, and segmentation. This design introduces computational overhead and complexity, which may hinder real-time applications. Furthermore, the authors did not incorporate advanced attention mechanisms, potentially limiting the ability to focus on relevant features effectively.

On a different approach, the integration of convolutional and transformer-based models in [26] and [27] both highlight the importance of leveraging transformers in medical image analysis. Still, optimizing the combination and interaction between these components and addressing issues related to computational complexity is crucial for achieving the best performance. Additionally, Cassidy et al. [28] proposed an Enhanced Harmonic Densely Connected Hybrid Transformer Network for chronic wound segmentation, utilizing multi-color space tensor merging to improve feature learning. Their work showed significant improvements in Dice scores for wounds on darker skin tones, addressing an important gap in wound segmentation. However, the added complexity of the hybrid transformer architecture could pose challenges for computational efficiency and scalability, especially in resource-limited settings like mobile health applications.

Other authors tried to address data challenges to enhance the robustness of the segmentation models. Galdran et al. [29] addressed the challenge of out-of-distribution samples by exploring the optimal combination of cross-entropy and soft Dice losses for lesion segmentation, demonstrating enhanced performance against unseen data. Hresko et al. [30] introduced a refined mixup augmentation strategy that outperforms traditional mixup augmentation and other data augmentation techniques, leading to improved segmentation results. Meanwhile, Brungel et al. [31] extended the dataset using synthetic data, enabling improved generalization capabilities. However, the quality of the generated synthetic data should be carefully addressed and evaluated.

### Attention Networks

Attention-based DL models have shown great potential in various computer vision tasks [32]. In the context of wound image segmentation, attention networks have been employed to effectively capture relevant features and focus on wound regions, offering improved accuracy of segmentation models.

The first paper to introduce attention networks specifically for medical image segmentation was Oktay et al. [7], who proposed the U-Net architecture that employs an attention gate mechanism for pancreas segmentation from CT images. Experimental results demonstrated improved segmentation accuracy compared to the standard U-Net model. A similar approach was proposed by Wang et al. [33] to perform brain tumor segmentation and experimental results supported the efficacy of adding an attention block to enhance Unet accuracy. The authors in [34], replaced Unet with a Residual UNet++ architecture obtained by adding residual units to a nested Unet architecture. The proposed approach improved segmentation accuracy on three different medical image datasets.

Another attention mechanism is to model channel-wise dependencies of the feature maps in a soft-attention manner using Squeeze and Excitation Networks (SENets) [8]. SENets have been successfully applied to various medical imaging modalities when combined with Unet variants, improving segmentation accuracy, and highlighting relevant structures [35–37]. Liang et al. [38] introduced RSEA-Net, integrating residual and SE blocks, which enhanced feature recalibration, significantly improving segmentation accuracy across various medical datasets.

Chae et al. [39] proposed a Residual U-Net architecture tailored for pressure ulcer segmentation. While effective, their model is computationally demanding, which limits its practicality for deployment in resource-constrained environments. Additionally, Chae et al. incorporated SE and attention blocks, but their lack of layered recalibration constrained the model’s ability to capture fine-grained details in deeper layers. Moreover, as the model was specifically designed for pressure ulcer segmentation, it risks weaker generalization to other wound types with diverse characteristics.

A notable recent contribution is the MedSAM paper [40], which introduced a specialized deep learning architecture with a self-attention mechanism for segmenting various medical images. MedSAM requires the selection of a bounding box for the region of interest (ROI), helping the model focus on specific areas and improving segmentation accuracy. While MedSAM demonstrated state-of-the-art performance across various datasets and effectively handles class imbalance, its computational complexity necessitates further optimization for practical deployment.

While attention networks and squeeze and excitation mechanisms offer a promising opportunity to enhance the efficiency of medical image segmentation, there is a lack of research conducted specifically in the context of wound image segmentation.

## Proposed Method

The aim of this research is to investigate the effect of attention layers and transfer learning on the performance of wound segmentation. The models will be trained and tested on different datasets to evaluate their ability to generalize. In this section, we introduce the techniques used in the Dual attention U-Net, beginning with the baseline VGG16-Unet model, followed by associated spatial and channel attention modules. An overview of the proposed architecture is presented in Fig. 1.

### Overview of the Baseline VGG16-UNet

The baseline model integrates two widely used architectures, VGG16 and U-Net, for image recognition and segmentation tasks. The encoder network from VGG16 and the decoder network from U-Net are combined to provide the benefits of both architectures. U-Net is a popular deep learning architecture designed for image segmentation tasks, consisting of an encoder network that captures context information and a decoder network that performs segmentation based on encoded features.

**Fig. 2:**
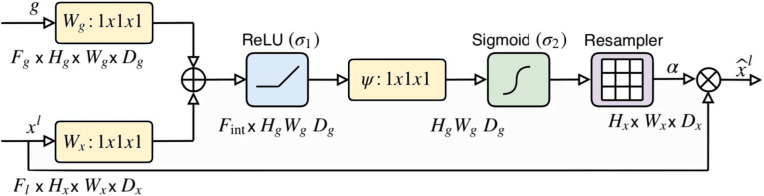
The spatial attention module schematic, where g is the gating signal, $x^l$ is the input feature map, $\bigoplus$ denotes add, and $\bigotimes$ denotes the elementwise multiplication. Figure reproduced from [7]

VGG16 is a popular convolutional neural network (CNN) that serves as a baseline architecture for many computer vision tasks [9]. It was trained on the ImageNet dataset, a large-scale image recognition dataset with over 14 million images in more than 20,000 classes. By using VGG16 as a backbone for U-Net and pre-trained weights from VGG16, the network achieves better performance (see the “Results and Discussion” section) and faster convergence, especially when working with limited data [41, 42].

In VGG16-U-Net, the VGG16-based encoder extracts high-level features from the input wound image, while the U-Net-based decoder performs pixel-wise segmentation. Also, the VGG16 model is modified to include skip connections to the decoder network, which helps preserve fine-grained details during the segmentation process.

The convolutional blocks in the encoder network consist of two or more consecutive 3 × 3 convolutions, followed by a ReLU activation function and a 2 × 2 max-pooling operation. In the decoder network, the convolutional blocks are similar to those used in U-Net, consisting of a 2 × 2 up-convolution (also known as transposed convolution), followed by concatenation along the channel dimension with the corresponding feature map from the encoder network. The concatenated feature maps are then passed through two or three consecutive 3 × 3 convolutions with ReLU activation, depending on the layer. Finally, skip connections connect the corresponding encoder and decoder layers to preserve spatial information during the up-sampling process.

### Dual Attention Pipeline

Attention mechanisms in neural networks refer to a way of selectively focusing on parts of the input during the processing of a neural network. The idea is inspired by the human brain’s ability to selectively process specific regions of input stimuli based on their relevance to the current task goals. In the context of semantic segmentation, attention mechanisms are used to selectively focus on specific regions of an image that are most relevant for segmentation [43, 44]. The idea is to assign a weight to each pixel in the feature maps based on its importance for the final segmentation output. Pixels with high weights are given more attention during the segmentation process, while pixels with low weights are given less attention.

One popular approach for incorporating attention mechanisms in semantic segmentation is the use of a spatial attention module so-called attention gate (AG). This module takes two inputs, the feature map from the encoder network at that level and the generated decoder output after an attention gate has been applied at the previous level, then generates a set of attention maps that indicate the importance of different regions in the feature map for segmentation. These attention maps are computed by passing the input image through a CNN and then applying a sigmoid activation function to the resulting feature map. The obtained maps are then used to selectively combine features from different regions of the feature map during the decoding process through an element-wise multiplication. This allows the decoder network to focus on the most relevant regions of the feature map for segmentation, while ignoring regions that are less relevant. The structure of the additive attention gate introduced in [7] is illustrated in Fig. 2.

Another approach for incorporating attention mechanisms in semantic segmentation is the use of a channel attention module. This module takes as input the feature map from the encoder network and generates a set of attention vectors that indicate the importance of different channels in the feature map for segmentation. The attention vectors are then used to selectively scale the features in each channel during the decoding process. This allows the decoder network to focus on the most relevant channels of the feature map for segmentation, while suppressing channels that are less relevant.

**Fig. 3:**
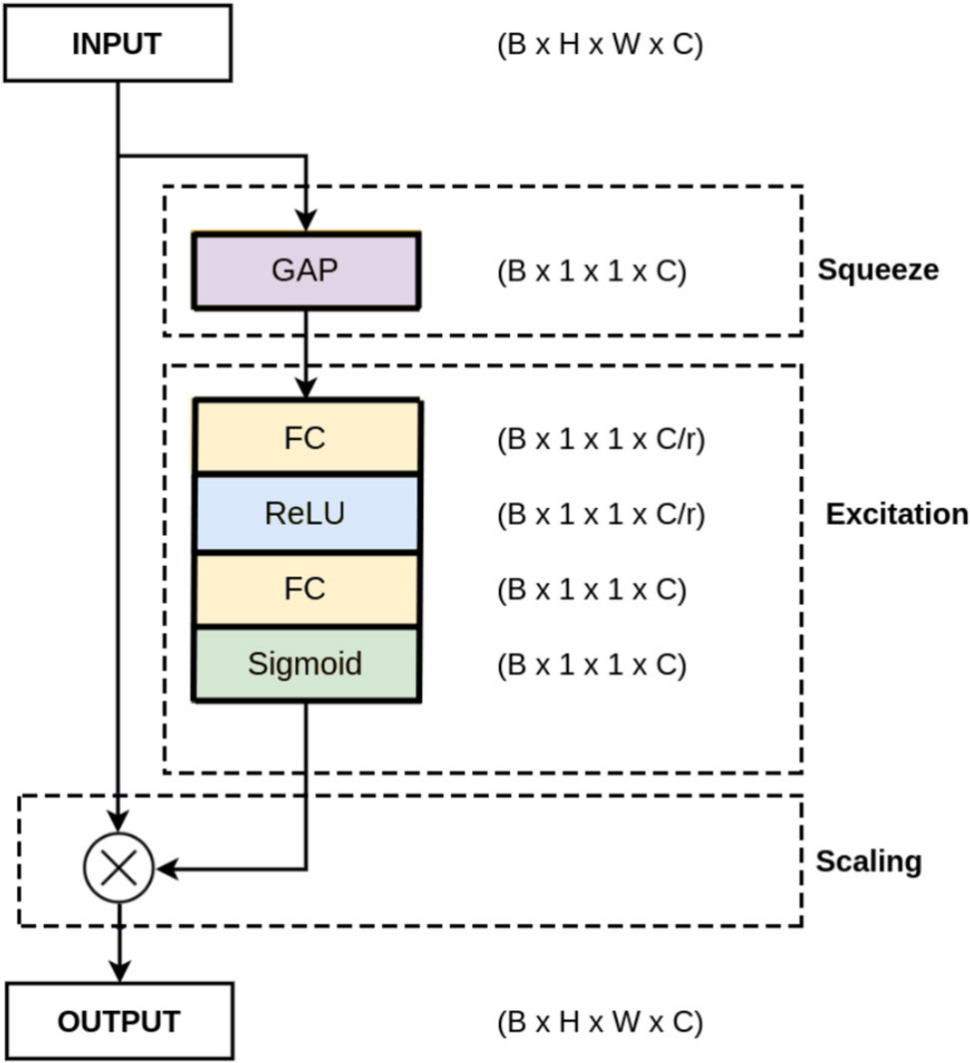
The channel attention module schematic, where C refers to the number of channels and r is the reduction ratio

Squeeze-and-Excitation (SE) is a type of attention mechanism that adaptively recalibrate the feature maps based on their channel-wise relevance to the target task [45]. The SE mechanism consists of two main components: the squeeze operation and the excitation operation. The squeeze operation is used to compress the spatial dimensions of the feature maps, reducing them to a global descriptor vector. This is achieved by applying a global average pooling operation over the feature maps along the spatial dimensions. The resulting vector represents a summary of the channel-wise features. The excitation operation is then used to generate channel-wise weights based on the learned importance of each feature map channel. This is achieved by passing the global descriptor vector through two fully connected layers with ReLU and sigmoid activations, which learn to generate a set of channel-wise weights [8, 46]. These weights are then applied to the original feature maps to highlight important channels and suppress irrelevant ones. The architecture of the channel attention module is shown in Fig. 3.

**Fig. 4:**
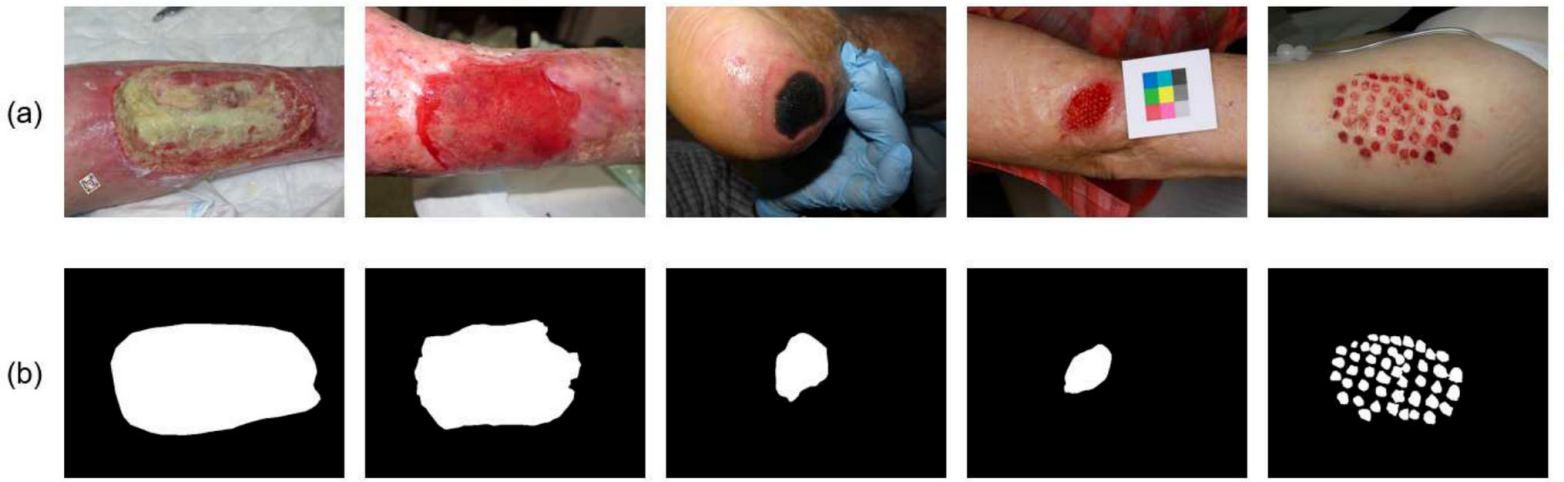
Some images from our database (**a**) and corresponding ground truth (**b**)

By adding SE blocks after each encoder and decoder, the network is able to perform context detection at different levels of abstraction. The SE blocks after the earlier encoders can detect coarse-grained context, while the SE blocks after the deeper encoders and decoders can detect fine-grained localization. This approach allows the network to make better feature selection, resulting in better segmentation performance.

## Databases

In this study, we utilized three distinct chronic wounds databases, as outlined below:

### WTS Dataset

The Wound Tissue Segmentation dataset (WTS) was collected for the purpose of this study. It is composed of images from multiple sources to ensure the representation from various wound pathologies and types. The collected wounds could be chronic or acute. The chronic ones include diabetic foot ulcers, pressure ulcers, venous ulcers, mixed ulcers, and arterial ulcers. On the other hand, acute wounds include burns, traumatic wounds, and surgical wounds, including skin grafts. This dataset comprises six skin tones according to the Fitzpatrick scale [47]. The data collection procedure was approved by Geneva’s research ethics committee (decision N°2022-00827) and all participants gave their consent to share their wound images.

For data diversity, wound images from two public databases were included [48, 49]. Consent for inclusion in the WTS was granted by the authors. The dataset consists of a total of 2230 images, each accompanied by corresponding annotations. It was then partitioned into training, validation, and test sets with a 70%, 20%, and 10% split ratio, respectively. The distribution of wound types and sizes was carefully considered to ensure a comprehensive representation.

**Table 1:** Wound pixel distribution across dataset subsets

Dataset subset	% of total data	Total wound pixels	Mean pixels/image	Max pixels/image	Min pixels/image
Training set	70%	21,718,410	13,922	161,154	0
Validation set	20%	6,200,854	13,780	142,694	0
Test set	10%	3,018,539	13,721	103,491	0

The process of labeling the images was conducted by a wound care specialist nurse who annotated the wound area and the tissue type (granulation, slough, necrosis, and epithelialization) by indicating their contours. Only the wound area is used as ground truth in this study. To validate the accuracy of labeling, a dermatologist carefully reviewed the annotations. This rigorous annotation process ensures the dataset’s reliability and makes it a valuable resource for training and evaluating AI models for wound recognition and analysis. Figure 4 provides visual examples of the wound annotations from the training set.

The dataset’s diversity is a key strength, as it includes wound images of varying sizes, shapes, colors, stages of healing, and skin tones. This diversity poses challenges for accurate segmentation, which is crucial for real-world applications in clinical settings. By exposing the model to such diverse scenarios, it is better prepared to handle the complexities encountered during actual clinical use, including home care.

### DFUC Dataset

The Diabetic Foot Ulcer Segmentation Challenge dataset (DFUC) was released at the 2022 MICCAI conference [19] and comprises 2000 images for training and 2000 images for testing, featuring pictures of both DFU and other foot conditions acquired with close-ups of the full foot using three different cameras. However, only the training set was released with corresponding ground truth segmentation masks. In this study, we divided the available data into our own training (70%) and validation (30%) splits.

### STANDUP Dataset

STANDUP dataset [15] comprises 569 images acquired from two sites, the Hospital Nacional Dos de Mayo in Lima, Peru, and the CHRO Hospital in Orleans, France. The photographs were taken following a practical and straightforward image acquisition protocol designed for clinical settings. This protocol involved capturing a single image of the wound area using a smartphone camera from a frontal perspective, under suitable room lighting, and without flash. The collected dataset showcases wounds of different sizes and are in various stages of healing. The entire database was used as a test set in the current study.

### FuSeg Dataset

The Foot Ulcer Segmentation Challenge (FuSeg) was organized in conjunction with the 2021 International Conference on Medical Image Computing and Computer-Assisted Intervention (MICCAI) [50]. The FuSeg dataset consists of 1210-foot ulcer images collected over a period of two years from 889 patients. This dataset focuses on the segmentation of foot ulcers, particularly diabetic foot ulcers, and includes high-resolution images along with corresponding ground truth segmentation masks. The diversity of wound images in terms of size, shape, and texture makes this dataset valuable for testing the generalization capability of wound segmentation model

## Experimental Results and Discussion

### Implementation

The proposed wound segmentation model was developed using the Python programming language and implemented in the Keras deep learning framework. The dataset was split into training (70%), validation (20%), and test (10%) sets, ensuring balanced wound pixel distribution and avoiding data leakage by including only one image per patient. Input images and masks had a resolution of 640 × 480 pixels.

Table 1 demonstrates significant variability in wound sizes, with a maximum of 161,154 pixels in the training set, ensuring that both small and large wounds were adequately represented. Such diversity is crucial for effective model training and performance on unseen data.

One of the challenges in wound imaging segmentation lies in the notable difference in the number of pixels belonging to the wound region compared to the background region. This class imbalance can introduce bias during model training, leading to poor wound delineation. To mitigate this issue, we employed a weighted cross-entropy loss function.

**Table 2:** Performance comparison of U-Net backbones

Network	DICE	IoU
U-net	**83.40% ($1.80 \times 10^{-6}$)	**74.70% ($1.43 \times 10^{-5}$)
VGG16 U-Net	**86.90% ($2.83 \times 10^{-6}$)	**79.10% ($1.78 \times 10^{-5}$)
ResNet50 U-Net	**68.90% ($5.06 \times 10^{-6}$)	**61.00% ($2.72 \times 10^{-5}$)
MobileNetV2 U-Net	**85.50% ($2.73 \times 10^{-6}$)	**77.30% ($1.30 \times 10^{-5}$)
Inception U-Net	**77.70% ($1.99 \times 10^{-6}$)	**68.80% ($1.18 \times 10^{-5}$)
DenseNet U-Net	**78.10% ($6.48 \times 10^{-6}$)	**69.00% ($3.34 \times 10^{-5}$)
EfficientNet U-Net	**82.90% ($7.56 \times 10^{-7}$)	**73.90% ($3.20 \times 10^{-6}$)

**Significant at *p*-value < 0.01

The bolded values indicate the best performance

The model was trained using the Adam optimizer for optimization with a learning rate of 1E-05. Also, early stopping is applied to prevent overfitting. The training process was stopped if the validation loss did not decrease for 10 epochs. This helped to prevent the model from memorizing the training data and resulted in a better generalization ability of the model. A batch size of 2 was considered due to memory constraints. The same settings for optimizer, learning rate, and loss function were used for all models to ensure a fair comparison between all networks.

After training the models on the DFUC dataset, we conducted an evaluation to assess their performance on two other datasets which were not used during training: WTS and STANDUP. Each database represents a unique set of challenges. This evaluation was carried out to test the generalization ability of each network and determine which model performed the best on unseen data.

### Performance Metrics

The evaluation metrics selected to evaluate the segmentation performance of the proposed method are Dice Similarity coefficient (DSC) and Intersection over Union score (IoU).

- Dice coefficient: Also known as the F1-score, it is a commonly used metric in medical image analysis to quantify the degree of overlap between the segmented and the ground truth mask [51].
- IoU score: Also called Jaccard index, it measures the similarity between two sets of pixels, specifically the overlap between the predicted and ground truth segmentation masks [52]. The IoU is calculated as the ratio of the intersection of the predicted and ground truth segmentation masks to their union.

To assess the statistical significance of the observed differences in performance metrics, we conducted a detailed statistical analysis. First, a normality test was performed to determine whether the data followed a normal distribution, ensuring the appropriate statistical test was applied. Subsequently, a paired t-test was conducted to compare the Dice and IoU scores across methods. The analysis was performed at a significance level of 99% ($p-value < 0.01$), indicating that differences were unlikely to have occurred by chance.

### Results and Discussion

#### Backbone Architecture Evaluation

A thorough set of experiments was conducted to determine the most suitable backbone architecture for the wound segmentation task with U-Net. Various state-of-the-art backbone architectures were systematically evaluated including VGG16 [9], VGG19 [9], ResNet50 [53], Inception ResNet v2 [54], MobileNet [55], DenseNet [56], and EfficientNetB0 [57]. The mean values of Dice and IoU scores are reported in Table 2, along with the corresponding p-values from paired t-tests to establish statistical significance. Results marked with $$^{**}$$ indicate statistically significant differences (*p* < 0.01) compared to the baseline U-net.

Among the tested backbones, VGG16 and MobileNetV2 outperformed other architectures.VGG16 was chosen as the best backbone architecture based on its superior performance. However, MobileNet V2 reached a very close DICE and IoU score with approximately half less parameters than VGG16, which make it an ideal candidate for an application on mobile devices.

#### Ablation Experiments

To determine the impact of attention layers and transfer learning on wound segmentation performance, we conducted an analysis of various models. We compared the standard U-Net model against U-Net with a VGG16 backbone (VGG16 U-Net), VGG16 U-Net with a squeeze-and-excitation module (SE VGG16 U-Net), VGG16 U-Net with spatial attention gates (AG VGG16 U-Net), and our proposed dual attention network (SE AG VGG16 U-Net) including spatial (AG) and feature (SE) attention. Table 4 provides a summary of our wound segmentation results including mean DICE and IoU scores along with standard deviations ($\pm \sigma$). To extend our analysis, we conducted a cross-dataset evaluation to assess the generalization capability of our models across diverse wound datasets. This approach aims to identify potential limitations and benchmark the effectiveness of our model in real-world healthcare settings. Additionally, we recorded the number of parameters and execution time per epoch for each model, which are presented in Table 3.

**Table 3:** Number of parameters

Network	Params	Time per epoch (s)
U-net	31,032,840	139
VGG16 U-Net	25,862,402	79
SE VGG16 U-Net	25,892,354	87
AG VGG16 U-Net	26,123,558	93
SE AG VGG16 U-Net	26,153,510	97

**Table 4:** Wound segmentation results

Database	DFUC		STANDUP		FuSeg		WTS	
Network	DICE (%$\pm \sigma$)	IoU (%$\pm \sigma$)	DICE (%$\pm \sigma$)	IoU (%$\pm \sigma$)	DICE (%$\pm \sigma$)	IoU (%$\pm \sigma$)	DICE (%$\pm \sigma$)	IoU (%$\pm \sigma$)
U-net	**83.40± 0.15	**74.70± 0.23	**82.60± 0.15	**73.40± 0.22	**74.20± 0.24	**65.40± 0.32	**79.40± 0.18	**69.60± 0.25
	($8.12 \times 10^{-6}$)	($1.24 \times 10^{-5}$)	($1.3 \times 10^{-3}$)	($8.3 \times 10^{-3}$)	($1.31 \times 10^{-5}$)	($4.76 \times 10^{-5}$)	($5.34 \times 10^{-5}$)	($8.12 \times 10^{-6}$)
VGG16 U-Net	**86.90± 0.12	**79.10± 0.20	**86.70± 0.11	**78.50± 0.18	**87.40± 0.12	**79.70± 0.20	**84.80± 0.13	**76.00± 0.20
	($1.14 \times 10^{-5}$)	($4.43 \times 10^{-5}$)	($4.07 \times 10^{-5}$)	($3 \times 10^{-4}$)	($1.78 \times 10^{-5}$)	($9.34 \times 10^{-6}$)	($1.53 \times 10^{-6}$)	($9.34 \times 10^{-6}$)
SE VGG16 U-Net	**89.50± 0.10	**82.60± 0.16	**89.50± 0.09	**82.30± 0.15	**88.40± 0.11	**81.10± 0.18	**86.00± 0.12	**77.50± 0.19
	($5.09 \times 10^{-6}$)	($2.62 \times 10^{-5}$)	($2.10 \times 10^{-5}$)	($2 \times 10^{-4}$)	($1.89 \times 10^{-6}$)	($1.49 \times 10^{-5}$)	($1.89 \times 10^{-6}$)	($1.89 \times 10^{-6}$)
AG VGG16 U-Net	**89.00± 0.10	**81.80± 0.17	**90.20± 0.08	**83.30± 0.14	**89.20± 0.10	**82.10± 0.17	**84.30± 0.13	**75.40± 0.21
	($2.94 \times 10^{-6}$)	($1.34 \times 10^{-5}$)	($2.66 \times 10^{-5}$)	($2 \times 10^{-4}$)	($1.80 \times 10^{-6}$)	($1.32 \times 10^{-5}$)	($1.32 \times 10^{-5}$)	($1.32 \times 10^{-5}$)
SE AG VGG16 U-Net	**90.20± 0.09	**83.**50**± **0**.**15**	**92.**90**±**0**.**06**	**87.**40**± **0**.**11**	**89.**60**± **0**.**10**	**82.**70**± **0**.**16**	**88.**00**± **0**.**10**	**80.**20**± **0**.**17**
	($1.01 \times 10^{-5}$)	($8.31 \times 10^{-5}$)	($2.96 \times 10^{-7}$)	($8.87 \times 10^{-6}$)	($7.58 \times 10^{-7}$)	($6.35 \times 10^{-6}$)	($3.41 \times 10^{-7}$)	($3.41 \times 10^{-7}$)

$$^{**}$$Significant at *p*-value < 0.01

The bolded values indicate the best performance

The results reveal that utilizing VGG16 as a backbone architecture considerably enhances the performance of the standard U-Net model across all metrics. The VGG16 U-Net model showed a 5% improvement in IoU and a 4% increase in Dice coefficient on the STANDUP dataset. Additionally, there was an increase of 14% and 13% in IoU and Dice for the FuSeg dataset. The performance improvement is similar on the WTS and DFUC sets. Moreover, VGG16 U-Net requires fewer training parameters, leading to improved computational efficiency.

We also found that incorporating SE modules after each encoder-decoder block further improved performance without adding any additional computational cost. The proposed SE VGG16 U-Net achieved higher Dice and IoU than VGG16 U-Net for the four testing sets (a 4% and 5% IoU improvement for STANDUP and WTS, respectively). This underscores the efficacy of integrating SE modules for both performance and efficiency gains.

Moreover, introducing Attention Gates (AG) alone into the (VGG16 U-Net) architecture demonstrates a positive impact on segmentation results. The (AG VGG16 U-Net) achieves competitive results, surpassing the baseline U-Net on all four databases, including DFUC, STANDUP, FuSeg, and WTS. This indicates that introducing attention mechanisms, even without Squeeze-and-Excitation (SE) blocks, enhances the model’s ability to focus on relevant features during the segmentation process.

Comparing the results of the SE VGG16 U-Net and the AG VGG16 U-Net, it is evident that both attention mechanisms contribute positively to segmentation accuracy. The SE VGG16 U-Net achieves higher scores on datasets like DFUC and WTS, but the AG VGG16 U-Net remains competitive across all datasets. This suggests that while SE blocks provide an additional level of feature recalibration, AG gates alone are effective in capturing relevant information for wound segmentation.

Our experimental results demonstrate that the combination of spatial and feature attention remarkably improves the performance of our proposed method, achieving the highest performance. Specifically, on the DFUC set, our method achieved a Dice coefficient of 90.2% and an IoU of 83.5%, indicating highly accurate segmentation. Moreover, the results on STANDUP show that the proposed dual attention method exhibits improved generalization with an increase of over than 5% for the IoU score and 3% for the Dice coefficient, compared to the single attention model with (SE). On the FuSeg dataset, similar performance improvements were observed, validating the robustness of the dual attention approach. Furthermore, in comparison to standard U-Net, the overall improvement was of 9% for the IoU score and 7% for the Dice coefficient for DFUC and 14% and 10%, respectively for STANDUP. Similar trends were observed in the WTS database with an improvement of 11% for IoU and 9% for Dice. These improvements are statistically significant ($p < 0.01$), suggesting that the dual attention mechanism effectively enhances the model’s ability to focus on relevant regions. The minimal variation in standard deviations underscores the robustness of the method. These results indicate that incorporating the attention gates played a critical role in enhancing the segmentation performance and generalization ability of the single attention model (SE VGG16 U-Net). Additionally, the computational cost of adding the AG module is still relatively small compared to the overall computational cost of training a standard U-Net network.

**Fig. 5:**
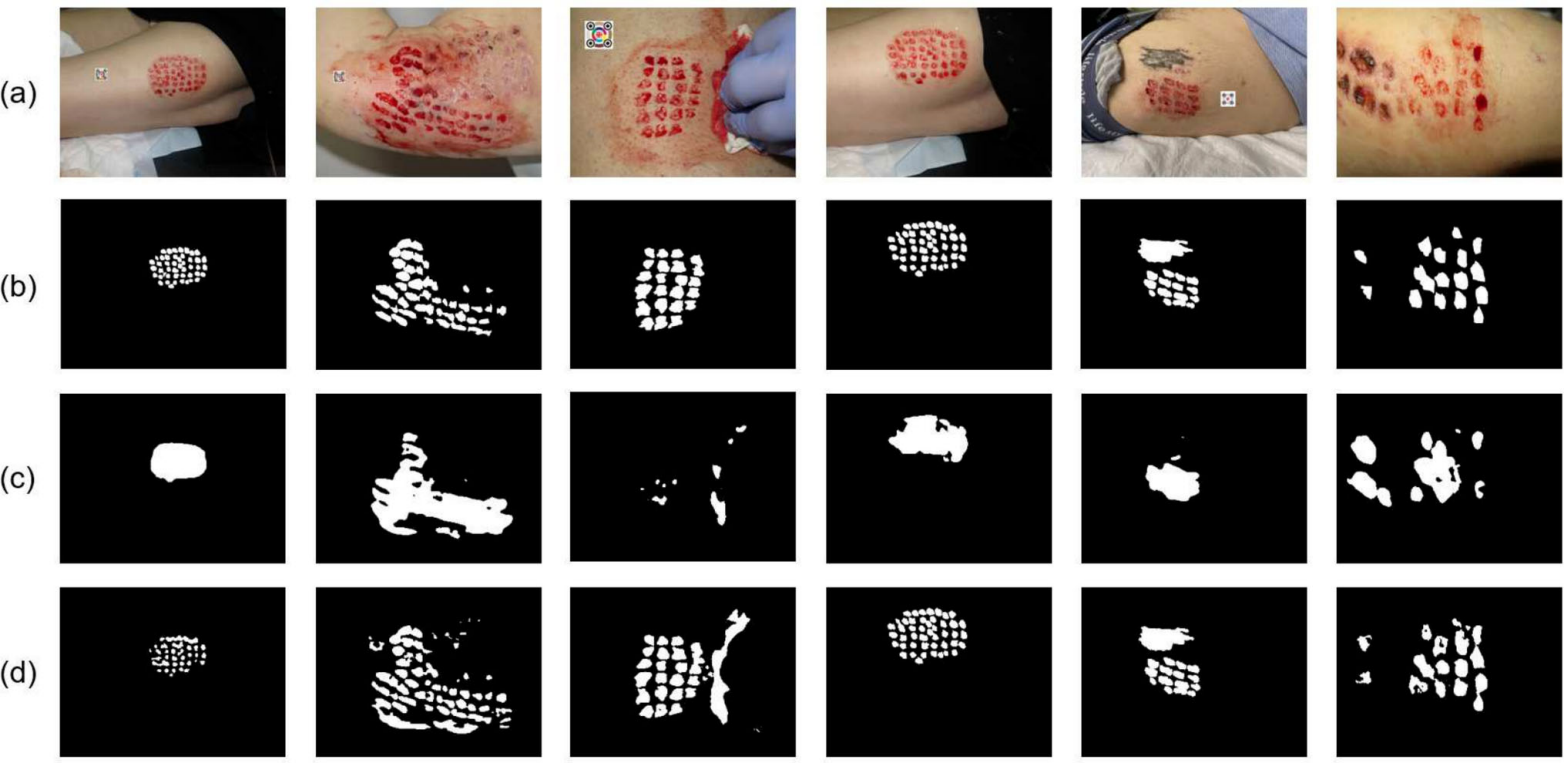
Segmentation results on WTS dataset: **a** original image, **b** ground truth, **c** predicted mask before fine-tuning, and **d** after fine-tuning

#### Generalization Experiments

To assess the generalization capability of our models across diverse wound datasets, we conducted cross-dataset evaluations. The aim was to identify potential limitations and benchmark the effectiveness of our model in real-world healthcare settings.

Despite the overall high performance of our model, differences in performance were observed between WTS and the other databases, as indicated in Table 4. A notable challenge emerged in WTS, where donor sites in skin grafts were erroneously considered as a single wound during segmentation as can be seen in Fig. 5c.

To address this issue, we opted for fine-tuning the model architecture to better align with the characteristics of our WTS database. The training was performed on the training and validation sets described in the “WTS Dataset” section. The refined model now successfully and precisely segments donor sites for skin grafts, rectifying the earlier misconception of grouping them as a single entity as illustrated in Fig. 5 and reported in Table 5. The presented outcomes are derived from evaluations conducted on the test set. This adaptation demonstrates the model’s flexibility and capacity for improvement, refining our approach for optimal results in a clinical setting. When benchmarked against state-of-the-art methods, including MedSAM, our Dual U-Net achieved the highest performance metrics (94.10% Dice and 89.30% IoU), underscoring its robustness. Additionally, the p-values from the paired t-tests confirmed that these results were statistically significant ($p-values<< 0.01$), reinforcing the robustness of our Dual U-Net.

**Table 5:** Wound segmentation results on WTS after fine-tuning, compared to the state of the art

Network	DICE (%$\pm \sigma$)	IoU (%$\sigma$)
U-net [6]	**84.40± 0.13	**75.50± 0.20
	($1.80 \times 10^{-6}$)	($1.43 \times 10^{-5}$)
DeepLabv3+ [58]	**77.10± 0.21	**67.80± 0.29
	($6.36 \times 10^{-5}$)	($2.66 \times 10^{-4}$)
Segnet [59]	**88.90± 0.09	**81.50± 0.15
	($2.89 \times 10^{-6}$)	($2.06 \times 10^{-5}$)
LinkNet [60]	**91.70± 0.07	**85.60± 0.12
	($3.43 \times 10^{-6}$)	($3.19 \times 10^{-6}$)
Hardnet-DFUC [21]	**92.61± 0.10	**89.10± 0.12
	($3.41 \times 10^{-7}$)	($2.36 \times 10^{-7}$)
MedSAM [40]	**78.16± 0.20	**73.14± 0.24
	(0.0)	($$1 \times 10^{-4}$$)
Dual U-Net (Ours)	** **94**.**10**± **0**.**05**	** **89**.**30**± **0**.**09**
	($1.39 \times 10^{-7}$)	($1.56 \times 10^{-6}$)

$$^{**}$$Significant at *p*-value < 0.01

The bolded values indicate the best performance

**Fig. 6:**
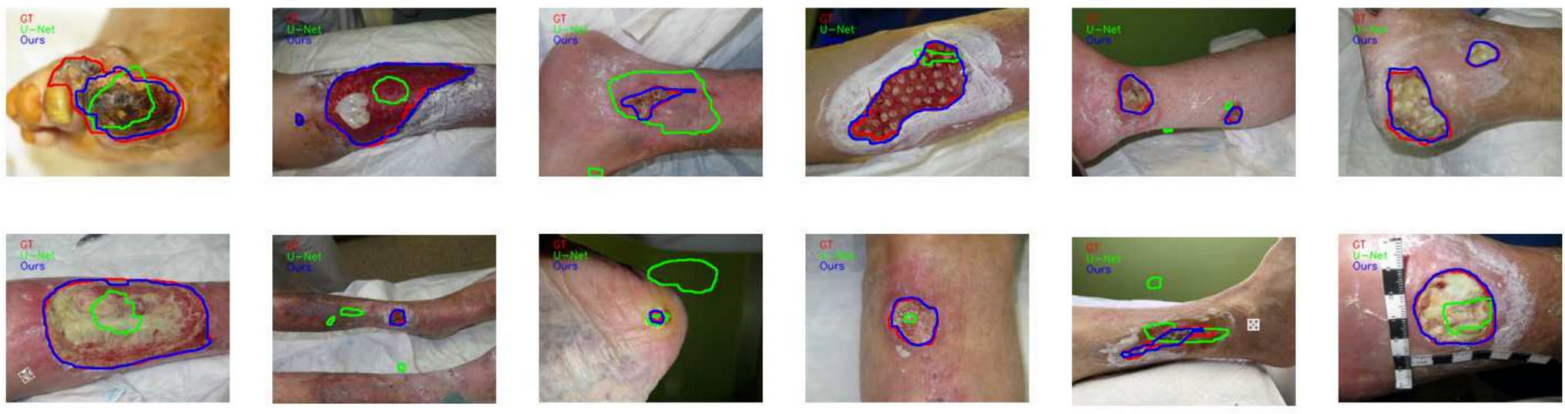
Qualitative results: Red, green, and blue contours represent ground truth, the outputs of U-Net, and the proposed Dual Attention network, respectively

**Fig. 7:**
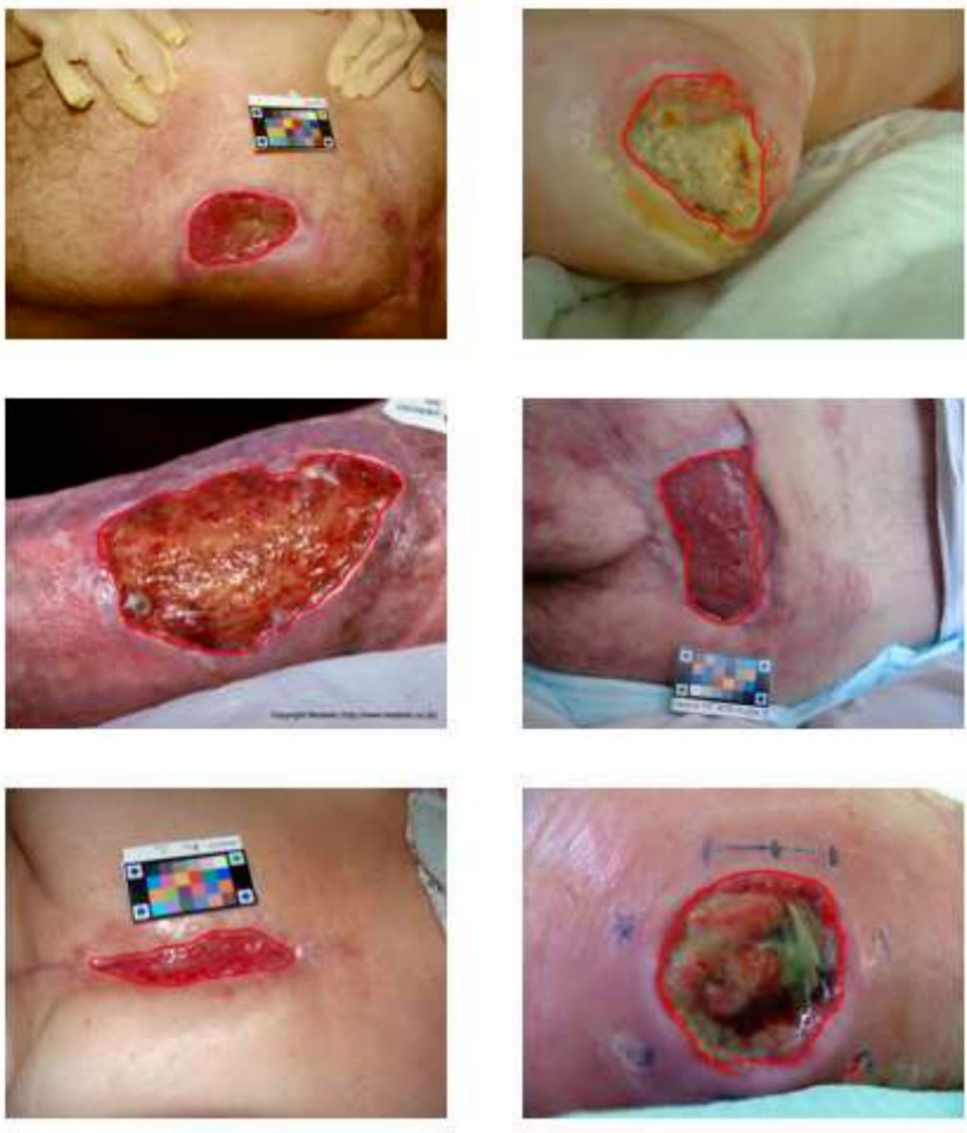
Segmentation results of the proposed method on STANDUP dataset

Figure 6 provides a comparison of the segmentation contours generated by our proposed dual attention model and the standard U-Net in comparison to the ground truth, using selected images from the WTS test set. As shown, our method exhibits a superior ability to focus on the wound area, leading to a more robust and reliable segmentation. Additionally, qualitative generalization performance on STANDUP is shown in Fig. 7. The results obtained demonstrate the generalizability of our proposed method. It shows impressive performance not only on unseen data, but also on other wound types that are larger and more complex. This highlights the ability of our method to accurately segment various types of wounds, which is a critical requirement for practical clinical applications.

#### Discussion

In summary, our dual attention model demonstrates state-of-the-art performance in wound segmentation, surpassing the classical U-Net architecture with fewer parameters, shorter training times, and superior accuracy. The metrics across multiple databases were particularly impressive, highlighting the model’s capacity to generalize effectively to diverse wound types and datasets. Qualitative results further emphasize this strength, as the segmentation contours closely approximate the ground truth. The ability to accurately segment unseen wound types underscores the model’s robustness and generalizability, which are critical for practical clinical applications.

In addition to the comparisons in the results section, our Dual attention model offers significant advantages over other SOTA methods. Chae et al. [39] employed a Residual U-Net with attention mechanisms for pressure ulcer segmentation, but their approach was computationally intensive and tailored to a single wound type. In contrast, our model leverages a VGG16-based encode, reducing parameters and training time while maintaining high accuracy. Furthermore, the inclusion of SE blocks at each encoder and decoder layer enables multi-level feature recalibration, improving the model’s ability to capture both coarse and fine-grained features. Our model also addresses limitations in [25], who used a multi-step segmentation process without attention mechanisms. By integrating dual attention mechanisms directly, our model reduces complexity while improving accuracy. As shown in the results section, it achieved an IoU of 82.7% on the FuSeg dataset, surpassing Scebba et al.’s 80%, demonstrating the efficiency and superiority of our design.

The effectiveness of the proposed method can be attributed to the incorporation of the dual attention mechanism, which enables it to identify and focus on the wound area, leading to improved segmentation performance. In addition, our method is more computationally efficient, making it a more cost-effective solution for wound segmentation in clinical settings. In conclusion, the proposed dual attention network is highly effective, achieving state-of-the-art performance in terms of accuracy, precision, and generalization. The results demonstrate its potential for practical clinical applications, such as wound monitoring and treatment evaluation.

Despite the advantages of our dual attention model for wound segmentation, several limitations must be acknowledged. While our model demonstrated strong performance across four external databases, the training dataset includes images representing six different skin tones, enhancing its applicability. However, it may still not fully encompass the diverse population of wound patients, which could affect the generalizability of our results. A validation study involving a larger, more ethnically diverse cohort is essential to establish the robustness of our model across varying skin types and wound conditions. Additionally, challenges in feature extraction can significantly impact the accuracy of wound segmentation, particularly when dealing with subtle wound characteristics. Imaging artifacts and lighting variations can obscure essential features, making it difficult for the model to differentiate between wounds and surrounding tissue. This limitation becomes particularly pronounced in cases of small non-open wounds, which may lack pronounced features and are often difficult to detect. As a result, the model may not detect actual wounds, leading to false negatives. This emphasizes the critical need for robust feature extraction methods that enhance detection capabilities and ensure reliable performance in clinical settings. Addressing these limitations through future research and refinement will be critical to improving the reliability and applicability of the model in real-world wound care scenarios. By comparing our approach to SOTA methods, we have demonstrated that the proposed dual attention network not only offers state-of-the-art performance but also provides practical advantages in terms of efficiency, accuracy, and generalization, reinforcing its value for clinical applications.

## Conclusion and Future Work

In this paper, we proposed a novel Dual attention VGG16-UNet model for wound image segmentation. The proposed model integrated the VGG16 and U-net architectures and included dual attention mechanisms that include attention gates and squeeze-and-excitation modules. By leveraging the high-level features extracted by the VGG16 architecture, the segmentation ability of the U-Net architecture has increased remarkably. Moreover, the inclusion of dual attention mechanisms further improved the model’s ability to focus on the relevant regions of the wound area, leading to improved segmentation accuracy. Our model achieved superior performance compared to other sota models, providing a promising solution to the challenge of wound segmentation and opens up new avenues for the development of more accurate and efficient wound analysis systems that can be used in clinical routine.

The proposed segmentation scheme can aid medical professionals in the monitoring and evaluation of wound healing over time, enabling faster wound management and more effective treatments. Further research can be carried out to investigate the transferability of the proposed model to different datasets. In collaboration with imito AG^1^ and the Haute Ecole de Santé of Geneva, we are collecting a large wound database using a customized Wound documentation App imitoWoundR. In this database, which will be available for research under the Open Science Initiative, we are collecting not only wound images, but also wound 3D meshes using the Structure Sensor,^2^ a video of the wound, the patient’s medical history, the wound status and the treatment. Furthermore, the potential of implementing the dual attention model on a smartphone and the development of a real-time wound segmentation system can be explored as potential future directions.

## Data Availability

The dataset generated and analyzed during the current study is not yet publicly available.

## Footnotes

1 https://imito.io/en

2 https://structure.io/

## References

[1] C. K. Sen, Human wound and its burden: updated 2020 compendium of estimates, *advances in wound care*, vol. 10, no. 5, pp. 281–292, 2021. 10.1089/wound.2021.0026 PMC8024242 33733885

[2] M. Edmonds, C. Manu, and P. Vas, “The current burden of diabetic foot disease,” *Journal of clinical orthopaedics and trauma*, vol. 17, pp. 88–93, 2021. 10.1016/j.jcot.2021.01.017 PMC7919962 33680841

[3] G. FrykbergRobert *et al.*, “Challenges in the treatment of chronic wounds,” *Advances in wound care*, 2015. 10.1089/wound.2015.0635 PMC4528992 26339534

[4] E. C. Montero, L. Atkin, M. Collier, A. Hogh, J. D. Ivory, K. Kirketerp-Moller, S. Meaume, H. Ryan, E. K. Stuermer, G.-S. Tiplica, *et al.*, “Lower leg ulcer diagnosis and principles of treatment,” *Journal of Wound Management*, vol. 24, no. 2, pp. S1–S75, 2023.

[5] C. T. Hess, “Comprehensive patient and wound assessments,” *Advances in Skin & Wound Care*, vol. 32, no. 6, pp. 287–288, 2019. 10.1097/01.ASW.0000558514.64758.7f 31107271

[6] O. Ronneberger, P. Fischer, and T. Brox, “U-net: Convolutional networks for biomedical image segmentation,” in *International Conference on Medical image computing and computer-assisted intervention*, pp. 234–241, Springer, 2015.

[7] O. Oktay, J. Schlemper, L. L. Folgoc, M. Lee, M. Heinrich, K. Misawa, K. Mori, S. McDonagh, N. Y. Hammerla, B. Kainz, *et al.*, “Attention u-net: Learning where to look for the pancreas,” arXiv preprint arXiv:1804.03999, 2018.

[8] J. Hu, L. Shen, and G. Sun, “Squeeze-and-excitation networks,” in *Proceedings of the IEEE conference on computer vision and pattern recognition*, pp. 7132–7141, 2018.

[9] K. Simonyan and A. Zisserman, “Very deep convolutional networks for large-scale image recognition,” arXiv preprint arXiv:1409.1556, 2014.

[10] D. H. Keast, C. K. Bowering, A. W. Evans, G. L. Mackean, C. Burrows, and L. D’Souza, “Contents: Measure: A proposed assessment framework for developing best practice recommendations for wound assessment,” *Wound Repair and Regeneration*, vol. 12, pp. s1–s17, 2004. 10.1111/j.1067-1927.2004.0123S1.x 15230830

[11] C. Wang, X. Yan, M. Smith, K. Kochhar, M. Rubin, S. M. Warren, J. Wrobel, and H. Lee, “A unified framework for automatic wound segmentation and analysis with deep convolutional neural networks,” in *2015 37th annual international conference of the ieee engineering in medicine and biology society (EMBC)*, pp. 2415–2418, IEEE, 2015. 10.1109/EMBC.2015.7318881 26736781

[12] N. Silberman, D. Hoiem, P. Kohli, and R. Fergus, “Indoor segmentation and support inference from rgbd images,” in *European conference on computer vision*, pp. 746–760, Springer, 2012.

[13] M. Goyal, M. H. Yap, N. D. Reeves, S. Rajbhandari, and J. Spragg, “Fully convolutional networks for diabetic foot ulcer segmentation,” in *2017 IEEE international conference on systems, man, and cybernetics (SMC)*, pp. 618–623, IEEE, 2017.

[14] F. Li, C. Wang, X. Liu, Y. Peng, and S. Jin, “A composite model of wound segmentation based on traditional methods and deep neural networks,” *Computational intelligence and neuroscience*, vol. 2018, 2018. 10.1155/2018/4149103 PMC6000917 29955227

[15] R. Niri, E. Gutierrez, H. Douzi, Y. Lucas, S. Treuillet, B. Castaneda, and I. Hernandez, “Multi-view data augmentation to improve wound segmentation on 3d surface model by deep learning,” *IEEE Access*, vol. 9, pp. 157628–157638, 2021.

[16] A. Mahbod, G. Schaefer, R. Ecker, and I. Ellinger, “Automatic foot ulcer segmentation using an ensemble of convolutional neural networks,” in *2022 26th International Conference on Pattern Recognition (ICPR)*, pp. 4358–4364, IEEE, 2022.

[17] H. Gamage, W. Wijesinghe, and I. Perera, “Instance-based segmentation for boundary detection of neuropathic ulcers through mask-rcnn,” in *International Conference on Artificial Neural Networks*, pp. 511–522, Springer, 2019.

[18] S. Zahia, B. Garcia-Zapirain, and A. Elmaghraby, “Integrating 3d model representation for an accurate non-invasive assessment of pressure injuries with deep learning,” *Sensors*, vol. 20, no. 10, p. 2933, 2020. 10.3390/s20102933 PMC7294421 32455753

[19] C. Kendrick, B. Cassidy, J. M. Pappachan, C. O’Shea, C. J. Fernandez, E. Chacko, K. Jacob, N. D. Reeves, and M. H. Yap, “Translating clinical delineation of diabetic foot ulcers into machine interpretable segmentation,” arXiv preprint arXiv:2204.11618, 2022.

[20] Y.-H. Chen, Y.-J. Ju, and J.-D. Huang, “Capture the devil in the details via partition-then-ensemble on higher resolution images,” in *Diabetic Foot Ulcers Grand Challenge*, pp. 52–64, Springer, 2022.

[21] T.-Y. Liao, C.-H. Yang, Y.-W. Lo, K.-Y. Lai, P.-H. Shen, and Y.-L. Lin, “Hardnet-dfus: Enhancing backbone and decoder of hardnet-mseg for diabetic foot ulcer image segmentation,” in *Diabetic Foot Ulcers Grand Challenge*, pp. 21–30, Springer, 2022.

[22] C.-H. Huang, H.-Y. Wu, and Y.-L. Lin, “Hardnet-mseg: A simple encoder-decoder polyp segmentation neural network that achieves over 0.9 mean dice and 86 fps,” arXiv preprint arXiv:2101.07172, 2021.

[23] H. Yi, W. Xu, Z. Jiang, J. Gao, Q. Kang, Q. Lao, and K. Li, “Ocrnet for diabetic foot ulcer segmentation combined with edge loss,” in *Diabetic Foot Ulcers Grand Challenge*, pp. 31–39, Springer, 2022.

[24] V. Gupta, A. Gupta, N. Arora, and J. Garg, “Ocrnet - light-weighted and efficient neural network for optical character recognition,” in *2021 IEEE Bombay Section Signature Conference (IBSSC)*, pp. 1–4, 2021.

[25] G. Scebba, J. Zhang, S. Catanzaro, C. Mihai, O. Distler, M. Berli, and W. Karlen, “Detect-and-segment: A deep learning approach to automate wound image segmentation,” *Informatics in Medicine Unlocked*, vol. 29, p. 100884, 2022.

[26] M. Hassib, M. Ali, A. Mohamed, M. Torki, and M. Hussein, “Diabetic foot ulcer segmentation using convolutional and transformer-based models,” in *Diabetic Foot Ulcers Grand Challenge*, pp. 83–91, Springer, 2022.

[27] D. Kucharski, A. Kostuch, F. Noworolnik, A. Brodzicki, and J. Jaworek-Korjakowska, “Dfu-ens: End-to-end diabetic foot ulcer segmentation framework with vision transformer based detection,” in *Diabetic Foot Ulcers Grand Challenge*, pp. 101–112, Springer, 2022.

[28] B. Cassidy, C. Mcbride, C. Kendrick, N. D. Reeves, J. M. Pappachan, C. J. Fernandez, E. Chacko, R. Brüngel, C. M. Friedrich, M. Alotaibi, *et al.*, “An enhanced harmonic densely connected hybrid transformer network architecture for chronic wound segmentation utilising multi-colour space tensor merging,” arXiv preprint arXiv:2410.03359, 2024. 10.1016/j.compbiomed.2025.110172 40318494

[29] A. Galdran, G. Carneiro, and M. A. G. Ballester, “On the optimal combination of cross-entropy and soft dice losses for lesion segmentation with out-of-distribution robustness,” in *Diabetic Foot Ulcers Grand Challenge*, pp. 40–51, Springer, 2022.

[30] D. J. Hresko, J. Vereb, V. Krigovsky, M. Gayova, and P. Drotar, “Refined mixup augmentation for diabetic foot ulcer segmentation,” in *Diabetic Foot Ulcers Grand Challenge*, pp. 92–100, Springer, 2022.

[31] R. Brüngel, S. Koitka, and C. M. Friedrich, “Unconditionally generated and pseudo-labeled synthetic images for diabetic foot ulcer segmentation dataset extension,” in *Diabetic Foot Ulcers Grand Challenge*, pp. 65–79, Springer, 2022.

[32] M.-H. Guo, T.-X. Xu, J.-J. Liu, Z.-N. Liu, P.-T. Jiang, T.-J. Mu, S.-H. Zhang, R. R. Martin, M.-M. Cheng, and S.-M. Hu, “Attention mechanisms in computer vision: A survey,” *Computational visual media*, vol. 8, no. 3, pp. 331–368, 2022.

[33] S. Wang, L. Li, and X. Zhuang, “Attu-net: attention u-net for brain tumor segmentation,” in *International MICCAI Brainlesion Workshop*, pp. 302–311, Springer, 2021.

[34] Z. Li, H. Zhang, Z. Li, and Z. Ren, “Residual-attention unet++: A nested residual-attention u-net for medical image segmentation,” *Applied Sciences*, vol. 12, no. 14, p. 7149, 2022.

[35] J. Zhang, X. Lv, H. Zhang, and B. Liu, “Aresu-net: Attention residual u-net for brain tumor segmentation,” *Symmetry*, vol. 12, no. 5, p. 721, 2020.

[36] J. Wang, P. Lv, H. Wang, and C. Shi, “Sar-u-net: Squeeze-and-excitation block and atrous spatial pyramid pooling based residual u-net for automatic liver segmentation in computed tomography,” *Computer Methods and Programs in Biomedicine*, vol. 208, p. 106268, 2021. 10.1016/j.cmpb.2021.106268 34274611

[37] G. Prasanna, J. R. Ernest, S. Narayanan, *et al.*, “Squeeze excitation embedded attention unet for brain tumor segmentation,” arXiv preprint arXiv:2305.07850, 2023.

[38] S. Liang, T. Wang, C. Chen, H. Liu, C. Qin, and Y. Feng, “Rsea-net: Residual squeeze and excitation attention network for medical image segmentation,” *BMC Medical Imaging*, 2022.

[39] J. Chae, K. Y. Hong, and J. Kim, “A pressure ulcer care system for remote medical assistance: residual u-net with an attention model based for wound area segmentation,” arXiv preprint arXiv:2101.09433, 2021.

[40] J. Ma, Y. He, F. Li, L. Han, C. You, and B. Wang, “Segment anything in medical images,” *Nature Communications*, vol. 15, no. 1, p. 654, 2024. 10.1038/s41467-024-44824-z PMC10803759 38253604

[41] A. A. Pravitasari, N. Iriawan, M. Almuhayar, T. Azmi, I. Irhamah, K. Fithriasari, S. W. Purnami, and W. Ferriastuti, “Unet-vgg16 with transfer learning for mri-based brain tumor segmentation,” *TELKOMNIKA (Telecommunication Computing Electronics and Control)*, vol. 18, no. 3, pp. 1310–1318, 2020.

[42] A. Huang, Q. Wang, L. Jiang, and J. Zhang, “Automatic segmentation of median nerve in ultrasound image by a combined use of u-net and vgg16,” in *2021 IEEE International Ultrasonics Symposium (IUS)*, pp. 1–4, 2021.

[43] S. Woo, J. Park, J.-Y. Lee, and I. S. Kweon, “Cbam: Convolutional block attention module,” in *Proceedings of the European conference on computer vision (ECCV)*, pp. 3–19, 2018.

[44] J. Fu, J. Liu, H. Tian, Y. Li, Y. Bao, Z. Fang, and H. Lu, “Dual attention network for scene segmentation,” in *Proceedings of the IEEE/CVF conference on computer vision and pattern recognition*, pp. 3146–3154, 2019.

[45] Z. Gao, J. Xie, Q. Wang, and P. Li, “Global second-order pooling convolutional networks,” in *Proceedings of the IEEE/CVF Conference on computer vision and pattern recognition*, pp. 3024–3033, 2019.

[46] L. Rundo, C. Han, Y. Nagano, J. Zhang, R. Hataya, C. Militello, A. Tangherloni, M. S. Nobile, C. Ferretti, D. Besozzi, *et al.*, “Use-net: Incorporating squeeze-and-excitation blocks into u-net for prostate zonal segmentation of multi-institutional mri datasets,” *Neurocomputing*, vol. 365, pp. 31–43, 2019.

[47] T. B. Fitzpatrick, “The Validity and Practicality of Sun-Reactive Skin Types I Through VI,” *Archives of Dermatology*, vol. 124, pp. 869–871, 06 1988. 10.1001/archderm.124.6.869 3377516

[48] M. Kręcichwost, J. Czajkowska, A. Wijata, J. Juszczyk, B. Pyciński, M. Biesok, M. Rudzki, J. Majewski, J. Kostecki, and E. Pietka, “Chronic wounds multimodal image database,” *Computerized Medical Imaging and Graphics*, vol. 88, p. 101844, 2021. 10.1016/j.compmedimag.2020.101844 33477091

[49] S. Yang, J. Park, H. Lee, S. Kim, B.-U. Lee, K.-Y. Chung, and B. Oh, “Sequential change of wound calculated by image analysis using a color patch method during a secondary intention healing,” *PloS one*, vol. 11, no. 9, p. e0163092, 2016. 27648569 10.1371/journal.pone.0163092 PMC5029888

[50] C. Wang, A. Mahbod, I. Ellinger, A. Galdran, S. Gopalakrishnan, J. Niezgoda, and Z. Yu, “Fuseg: The foot ulcer segmentation challenge,” *Information*, vol. 15, no. 3, p. 140, 2024.

[51] L. R. Dice, “Measures of the amount of ecologic association between species,” *Ecology*, vol. 26, no. 3, pp. 297–302, 1945.

[52] K. Pearson, “Mathematical contributions to the theory of evolution.—on a form of spurious correlation which may arise when indices are used in the measurement of organs,” *Proceedings of the royal society of london*, vol. 60, no. 359-367, pp. 489–498, 1897.

[53] K. He, X. Zhang, S. Ren, and J. Sun, “Deep residual learning for image recognition,” in *Proceedings of the IEEE conference on computer vision and pattern recognition*, pp. 770–778, 2016.

[54] C. Szegedy, S. Ioffe, V. Vanhoucke, and A. A. Alemi, “Inception-v4, inception-resnet and the impact of residual connections on learning,” in *Thirty-first AAAI conference on artificial intelligence*, 2017.

[55] A. G. Howard, M. Zhu, B. Chen, D. Kalenichenko, W. Wang, T. Weyand, M. Andreetto, and H. Adam, “Mobilenets: Efficient convolutional neural networks for mobile vision applications,” arXiv preprint arXiv:1704.04861, 2017.

[56] G. Huang, Z. Liu, L. Van Der Maaten, and K. Q. Weinberger, “Densely connected convolutional networks,” in *Proceedings of the IEEE conference on computer vision and pattern recognition*, pp. 4700–4708, 2017.

[57] M. Tan and Q. Le, “Efficientnet: Rethinking model scaling for convolutional neural networks,” in *International conference on machine learning*, pp. 6105–6114, PMLR, 2019.

[58] L.-C. Chen, Y. Zhu, G. Papandreou, F. Schroff, and H. Adam, “Encoder-decoder with atrous separable convolution for semantic image segmentation,” in *Proceedings of the European conference on computer vision (ECCV)*, pp. 801–818, 2018.

[59] V. Badrinarayanan, A. Kendall, and R. Cipolla, “Segnet: A deep convolutional encoder-decoder architecture for image segmentation,” *IEEE transactions on pattern analysis and machine intelligence*, vol. 39, no. 12, pp. 2481–2495, 2017. 10.1109/TPAMI.2016.2644615 28060704

[60] A. Chaurasia and E. Culurciello, “Linknet: Exploiting encoder representations for efficient semantic segmentation,” in *2017 IEEE visual communications and image processing (VCIP)*, pp. 1–4, IEEE, 2017.

